# Inhibitory Effects of Astaxanthin on CML-HSA-Induced Inflammatory and RANKL-Induced Osteoclastogenic Gene Expression in RAW 264.7 Cells

**DOI:** 10.3390/biomedicines10010054

**Published:** 2021-12-27

**Authors:** A. N. M. Mamun-Or-Rashid, Tanzima Tarannum Lucy, Masayuki Yagi, Yoshikazu Yonei

**Affiliations:** Anti-Aging Medical Research Center and Glycative Stress Research Center, Graduate School of Life and Medical Sciences, Doshisha University, Kyoto 610-0394, Japan; amamun@mail.doshisha.ac.jp (A.N.M.M.-O.-R.); myagi@mail.doshisha.ac.jp (M.Y.); yyonei@mail.doshisha.ac.jp (Y.Y.)

**Keywords:** astaxanthin, glycation, CML-HSA, inflammation, RANKL, osteoclastogenesis, RAW 264.7 cells

## Abstract

Objective: Elevated levels of serum *N^ε^*-carboxymethyllysine (CML), a well-known advanced glycation end-product (AGE), were observed in patients with inflammation or osteoporosis. Astaxanthin was reported to possess anti-inflammatory and antioxidant effects. In the present study, we investigated the effects of commercially available dietary supplement AstaReal ACT^R^ (ASR) capsule content as astaxanthin on CML-HSA-induced inflammatory and receptor activator of nuclear factor-kappa-Β ligand (RANKL)-induced osteoclastogenic gene expression. Methods: RAW 264.7 murine macrophage cells were stimulated with CML-HSA to trigger inflammatory gene expression and treated with either a vehicle control or varied concentrations of astaxanthin. Inflammatory gene expression was measured using an enzyme-linked immunosorbent assay (ELISA) or qPCR. We triggered osteoclastogenesis using RANKL, and osteoclastogenic gene expression was measured through tartrate-resistant acid phosphatase (TRAP) activity, staining, immunofluorescence, and qPCR analyses. Results: CML-HSA showed a stimulatory effect on inflammatory gene expression, and astaxanthin reduced the expression by at least two-fold. The levels of autoinflammatory gene expression were reduced by astaxanthin. The RANKL-induced osteoclastogenesis was significantly inhibited by astaxanthin, with reductions in the activation of nuclear factor-κB (NF-κB), the expression of NFATc1 (nuclear factor of activated T cells 1), multinucleated cell formation, and the expression of mature osteoclast marker genes. Conclusion: Astaxanthin has potential as a remedy for CML-HSA-induced inflammation and RANKL-induced excessive bone loss.

## 1. Introduction

Inflammatory cytokines play a crucial role in inducing bone loss, resulting in bone diseases, such as osteoporosis [1], rheumatoid arthritis (RA) [2], osteoarthritis [3], periodontitis [4], and so on. The tumor necrosis factor alpha (TNFα) [5,6,7], Interleukin (IL)-1 family [8,9,10,11], and IL-6 [12,13,14,15] are reported to be directly or indirectly involved in inducing osteoclast formation and bone resorption by upregulating RANKL and other osteoclast-inducing factors that are usually secreted by osteoblasts and osteocytes and are expressed highly by the lymph nodes, thymus, and lungs and indigently by the spleen, bone marrow, peripheral blood, placenta, thyroid, leucocytes, stomach, skeletal muscle, and heart [16]. RANKL is a member of the family of tumor necrosis factors, which binds to its receptor RANK and activates different pathways to induce osteoclast differentiation. Inflammatory cytokines are also reported to stimulate RANKL-induced osteoclastogenesis [5,6,16,17,18,19,20], and some cytokines can induce osteoclastogenesis in the absence of RANKL [8,20,21]. Conversely, anti-inflammatory cytokines can downregulate osteoclastogenesis [22], providing evidence for the influential involvement of cytokines in bone remodeling. Bone remodeling is a lifelong fundamental phenomenon that characterizes bone as a dynamic tissue; it is comprised mainly of bone resorption by osteoclast cells and bone formation by osteoblast cells [23]. To maintain the integrity of the bone tissue, a balance between the activities of osteoblasts and osteoclasts is critical. However, it has been observed in osteoporosis-like diseases that bone resorption exceeds bone formation, leaving the bone weaker, fragile, and prone to fracture resulting from minor trauma [1].

Proteins react nonenzymatically with reactive dicarbonyls, glucose, and fructose in all living tissues and bodily fluids, referred to as glycation reaction, and this yields advanced glycation end-products (AGEs) as the final product, which is linked to the mechanism or progression of many age-related diseases as well as to the aging process [24,25,26,27,28,29,30]. AGE formation is a complex series of reactions starting with the glucose-mediated Schiff base formation, followed by formation of the intermediate Amadori product, which is then followed by several changes that ultimately result in the formation of AGEs. Accumulation of these AGEs is an inevitable component of the aging process in humans and other eukaryotes. Browning (a change occurring upon glycation that is visible), crosslinking, fluorescence at different wavelengths, and insolubility are major characteristics of the AGEs that are responsible for the irreversible structural and functional damages of biological macromolecules. The Baynes, Thorpe, and Monnier laboratories have significantly contributed to the volume of evidence concerning the link between the AGE accumulation and aging [31]. Nonenzymatic collagen crosslinking (i.e., collagen glycation) has been reported to be linked with arterial disease in patients with diabetes as well as in elderly people [32,33]. A cohort study in people 65 years or older revealed that increased *N^ε^*-carboxymethyllysine (CML) levels in plasma were positively correlated with a higher risk of mortality in elderly people with cardiovascular disease (CVD) and in all-cause mortality [34]. Furthermore, this association was independent of diabetes mellitus [34], indicating that AGE formation may not be dependent on glucose levels; instead, the structure of an individual’s proteins or their lifestyle may accelerate or decelerate AGE formation and accumulation. AGEs may not only act as important biomarkers but also have the potential to accelerate aging [35]. However, another study on a diabetic model of Goto-Kakizaki rats fed with free CML (2 mg/kg body weight) for 8 weeks revealed that consumption of exogenous free CML resulted in alterations in amino acids, carbohydrate metabolism, and the citric acid cycle that may be responsible for increased progression of diabetic disease and its complications. They also found an increase in oxidative stress levels, fasting blood glucose levels, altered values for beta cell function indices, and homeostasis model assessment in the serum and the urine of CML-fed rats compared with the control group [36].

AGEs are responsible for producing inflammation and oxidative stress by generating reactive oxygen species (ROS), reactive nitrogen species (RNS), and inducible nitric oxide synthase (iNOS) through activation of the nuclear factor-kappa B (NF-κB) pathway [36,37]. CML is a chief AGE, originating from the glycation reaction between the lysine of a protein and glucose or its derivatives. It was reported to be found in the synovial fluid of RA patients [38]. Another well-known AGE, pentosidine, was reported to have a detrimental effect on bone strength [39]. CML-HSA at concentrations of 0.5–2.0 µg/mL was reported to prompt TNFα secretion in an in vitro culture of RAW 264.7 cells, where TNFα secretion reached its highest level after 3 h of CML-HSA treatment and then declined [40], providing evidence for the involvement of TNFα secretion in inflammatory responses.

It is difficult to define the relationship between AGEs and age-related diseases for several reasons, including the slow accretion of AGEs; the diversity of sources; a flourishing number of targets of AGEs; the lack of a proper method for isolation, purification, identification, and quantification of specific AGEs in their native form from the mixture of thousands of AGEs; and a lack of models that replicate the pathologies resulting from the AGE accumulation. The aforementioned factors have made it difficult to create a model in which an AGE is linked to a single specific target and a single specific age-related disease [35]. However, AGEs demonstrate positive correlations with numerous chronic diseases that usually take years to manifest. Due to the high cost of treatment for chronic inflammatory diseases, natural products have consistently attracted interest for their use in treatment. Studies have shown that several plant extracts can significantly reduce the CML-HSA-induced TNFα production in RAW 264.7 cells [41]. Xanthophyll belongs to the family of oxygenated derivatives of carotenoid in which astaxanthin, a carotenoid pigment, also a member and is often found in various aquatic organisms, such as shrimp, crabs, red yeast, trout, salmon, algae, krill, and crayfish [42,43]. Carotenoids contain polyene chains and long conjugated double bonds that can reduce oxidative stress by quenching singlet oxygen and scavenging radicals to terminate their chain reactions. Naguib et al. reported that astaxanthin is a potent antioxidant in comparison to other carotenoids, such as lutein, lycopene, α-carotene, and β-carotene [44]. Studies on ethanol-induced gastric-ulcer rats and skin-cancer rats showed that astaxanthin and its esters exerted 80% antilipid peroxidation activity [45,46]. Ohgami et al. reported astaxanthin as a promising molecule for the treatment of ocular inflammation [47]. Astaxanthin was shown to prevent skin thickening and reduce collagen reduction in UV-induced skin damage [45,48]. It also shows potential as a therapeutic agent in the treatment of atherosclerotic cardiovascular disease [49]. Moreover, antidiabetic, anticarcinogenic, immune-modulating, and anti-gastric activities have also been reported [45,46,50]. Astaxanthin supplement at 3.6 mg per day may be beneficial for human health, according to a study by Iwamoto et al. [51].

Astaxanthin is lipophilic, heat sensitive, and difficult to preserve. Therefore, a special formulation is required for the maintenance of its structural and functional integrity. The collection, extraction, purification, and storage of astaxanthin requires proper care using specialized techniques [52,53,54,55,56,57]. AstaReal ACT^R^ (ASR) is a capsule formulation of astaxanthin sourced from *Haematococcus pluvialis*, which is a freshwater single-cell alga. It is essential to maintain the quality and functionality of astaxanthin for commercial production as a food supplement. In a previous study, research-grade astaxanthin (Sigma–Aldrich Co., St. Louis, MO, USA) was reported to inhibit osteoclast formation in an in vitro model of bone marrow cells that were isolated from the femur and tibia of 5-week-old male ICR mice and reduced bone loss in a C3H/HeN female murine model [58]. Therefore, the aim of this study was to investigate whether research-grade pure astaxanthin [58] and commercially available ASR supplements possessed the same effect on osteoclastogenesis. Moreover, having a multifunctional role, astaxanthin may have potential as a candidate for reducing inflammation, especially in AGE-induced inflammatory diseases. In the present study, we also investigated the impact of ASR supplements on CML-HSA-induced inflammatory gene expression and RANKL-induced osteoclastogenic gene expression in RAW 264.7 cells. As one of our previous studies reported that CML-HSA inhibited RANKL-induced osteoclastogenesis [59], akin to the effect of astaxanthin [60], we did not investigate the effect of astaxanthin on CML-HSA-induced osteoclastogenic gene expression.

## 2. Materials and Methods

### 2.1. Reagents and Chemicals

The commercially available astaxanthin supplement AstaReal ACT^R^ (ASR) (AstaReal Co., Ltd., Minato-ku, Tokyo, Japan) was used in our experiments. Each capsule contained 3.025 kcal, 0.135 g of protein, 0.265 g of lipid, 0.025 g of carbohydrate, and 0.05 mg of Na. The test product contained olive oil, gelatin, *Haematococcus pluvialis*, tocotrienol, glycerin, glycerol ester, beeswax, and L-ascorbic acid 2-glucoside.

Each capsule contains 6 mg of astaxanthin (3,3′-dihydroxy-β,β′-carotene-4,4′-dione). Dimethyl sulfoxide (DMSO) (Wako, Osaka, Japan) was used as the vehicle control, as it was used as a solvent to dissolve the ASR capsule contents. After being dissolved, the contents were diluted with culture media to acquire the mentioned concentrations of astaxanthin. Therefore, because of this processing, we use the term astaxanthin, instead of ASR, in the Section 3. DMSO was maintained at 0.2% (*v*/*v*) in all experiments. The CML-HSA was bought from CircuLex, Nagano, Japan. All other chemicals were analytical grade and were purchased from Sigma–Aldrich or Wako.

### 2.2. Cell Culture

RAW 264.7 cells (ATCC TIB-71), murine monocyte/macrophage cell lineage, were obtained from American Type Culture Collection (ATCC; Manassas, VA, USA). Dulbecco’s modified Eagle’s medium (DMEM) (Sigma–Aldrich, St. Louis, MO, USA) supplemented with heat-inactivated (HI) 10% (*v*/*v*) fetal bovine serum (FBS) (Nichirei Biosciences, Tokyo, Japan), 100 units/mL penicillin, 100 µg/mL streptomycin, and 25 µg/mL amphotericin B (Gibco, El Paso, TX, USA) was used as cell culture media, and incubation conditions were 37 °C temperature and 5% CO_2_ [40].

### 2.3. Cell Cytotoxicity

**Figure 1:**
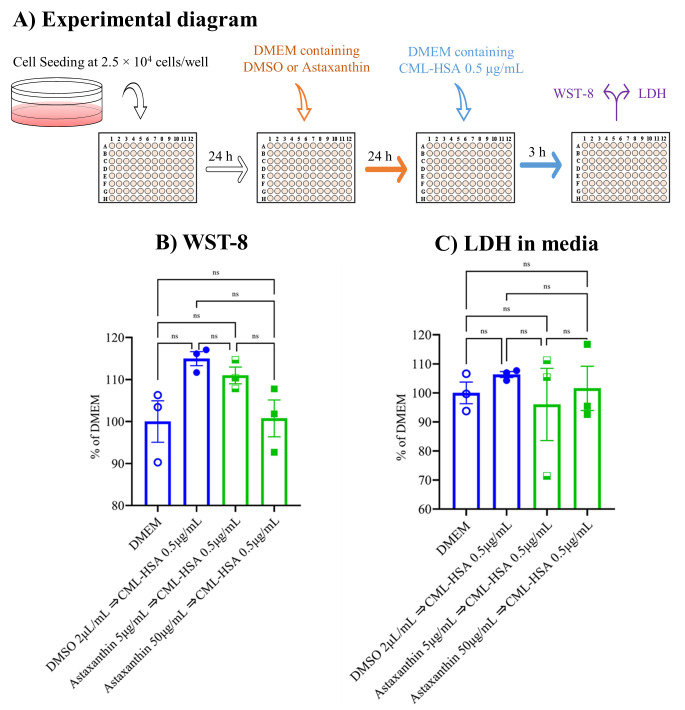
Effect of CML-HSA and astaxanthin on RAW 264.7 cell viability. (**A**) Schematic representation of experimental procedures. RAW 264.7 cells were seeded at 2.5 × 10^4^ cells/well in 96-well plate and pretreated with DMEM containing 10% FBS with or without astaxanthin (5 or 50 µg/mL) for 24 h. These cells were then treated with CML-HSA for 3 h. (**B**) WST-8 assay to determine cell viability. (**C**) Conditioned medium was used for LDH cytotoxicity assay. All data are shown as means ± SEM, *n* = 3, ns denoted not significant by Tukey–Kramer test. FBS, fetal bovine serum; DMEM, Dulbecco’s modified Eagle’s medium; DMSO, dimethyl sulfoxide; CML-HSA, *N^ε^*-carboxymethyllysine–human serum albumin; WST, water-soluble tetrazolium salt; LDH, lactate dehydrogenase; SEM, standard error mean.

The WST-8 and lactate dehydrogenase (LDH) assays were used to assess the viability of RAW 264.7 cells. The Cell Counting Kit 8 (CCK-8) was used for the WST-8 assay and performed according to the manufacturer’s protocol (Dojindo, Kumamoto, Japan). Briefly, RAW 264.7 cells were seeded at 2.5 × 10^4^ cells/well in 96-well plates for 24 h followed by treatment with the indicated concentrations of astaxanthin or DMSO for an additional 24 h. Afterwards, media was replaced with 0.5 µg/mL CML-HSA-containing media and was incubated for 3 h. After 2 h of CML-HSA treatment, 10 µL/well CCK-8 was added for WST-8 assay and the mixture was then incubated for another 1 h. Finally, after a total of 3 h of CML-HSA treatment (Figure 1A), or as otherwise indicated in Figure 4A, absorbance at 450 nm was measured by a Varioscan Flash microplate reader (Thermo Fisher Scientific, Waltham, MA, USA) [40,61]. The method described by Sato et al. was used to perform LDH assay [40]. To evaluate the effect of CML-HSA and astaxanthin on cell cytotoxicity, 50 µL of culture media from each well of the treated culture plate was collected and used for performing the LDH assay.

### 2.4. TNFα Measurement by ELISA

**Figure 2:**
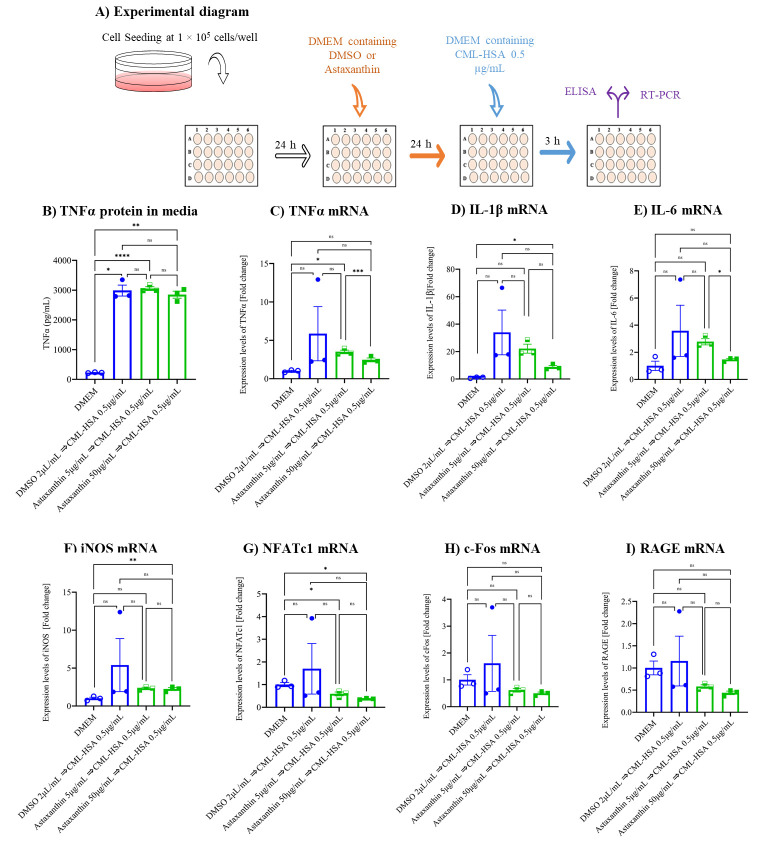
Effect of astaxanthin on CML-HSA-induced inflammatory gene expression. (**A**) Schematic representation of experimental procedures. Cells were seeded in Dulbecco’s modified Eagle’s medium (DMEM) at concentration of 1 × 10^5^ cells/well in 24-well plates and treated with either astaxanthin or DMSO for 24 h, followed by CML-HSA for an additional 3 h. (**B**) Secreted TNFα protein in media was determined by ELISA. qPCR analyses were performed for determining mRNA expression of (**C**) TNFα, (**D**) IL-1β, (**E**) IL-6, (**F**) iNOS, (**G**) NFATc1, (**H**) c-Fos, and (**I**) RAGE. All data were normalized using GAPDH and are shown as the mean ± SEM (*n* = 3) of the ratios against no treatment. * *p* < 0.05, ** *p* < 0.01, *** *p* <0.001, **** *p* <0.0001, ns denoted not significant by Tukey–Kramer test. CML-HSA, *N^ε^*-carboxymethyllysine–human serum albumin; TNFα, tumor necrosis factor alpha; DMSO, dimethyl sulfoxide; IL, interleukin; iNOS, inducible nitric oxide synthase; RAGE, receptor for advanced glycation end-products; NFATc1, nuclear factor of activated T-cell, cytoplasmic 1; GAPDH, glyceraldehyde-3-phosphate dehydrogenase; SEM, standard error mean.

Cells were seeded and treated with astaxanthin or DMSO for 24 h, which was followed by changing the medium to DMEM containing 0.5 µg/mL CML-HSA for 3 h (Figure 2A). Subsequently, culture media were collected and briefly centrifuged to settle the cells. The supernatant media were transferred into new vials and stored at −60 °C until use. TNFα concentration in the cell culture medium was measured using ELISA (mouse TNFα ELISA kit, ab100747; Abcam, Cambridge, UK), which was performed according to the manufacturer’s protocol. Absorbance was recorded at 450 nm using a Varioscan Flash microplate reader. A standard curve was prepared using recombinant mouse TNFα of known concentrations (93.75–6000.00 pg/mL) for calculating the TNFα concentrations in the experimental culture media [61].

### 2.5. In Vitro Osteoclastogenesis

RAW 264.7 cells were seeded at aforementioned cell density in multiwell chambers and incubated for 24 h. The media were then replaced with αMEM (Gibco) supplemented with 10% HI-FBS, antibiotics, and 100 ng/mL recombinant mouse RANK Ligand (rmRANKL, R&D Systems, Minneapolis, MN, USA) [62] with either DMSO or astaxanthin. After three days of incubation, the culture medium was renewed. After five days, the cells were subjected to various assays (Figure 4A).

### 2.6. TRAP Activity

After the osteoclastogenesis experiment was completed, as mentioned above and shown in Figure 4A, cell fixation buffer (acetone:ethanol = 1:1) was used to fix the cells, and a TRAP solution kit (Oriental Yeast Co., Tokyo, Japan) was then used to measure TRAP activity according to the manufacturer’s instruction. Afterwards, absorbance at 405 nm using a Varioscan Flash microplate reader (Thermo Fisher Scientific, Waltham, MA, USA) was measured [63,64].

### 2.7. TRAP Staining

Treated cells (Figure 4A) were fixed using a 10% formalin neutral buffer solution and a TRAP staining kit (387A-1KT, Sigma–Aldrich, USA) was used to stain fixed cells according to the manufacturer’s protocol. Multinucleated cells with ≥4 or ≥10 nuclei were counted as small and giant osteoclast cells, respectively, under a light microscope (CKX 41, Olympus, Tokyo, Japan), and representative images were captured [60,63].

### 2.8. Isolation of Total RNA and RT-PCR

**Figure 3:**
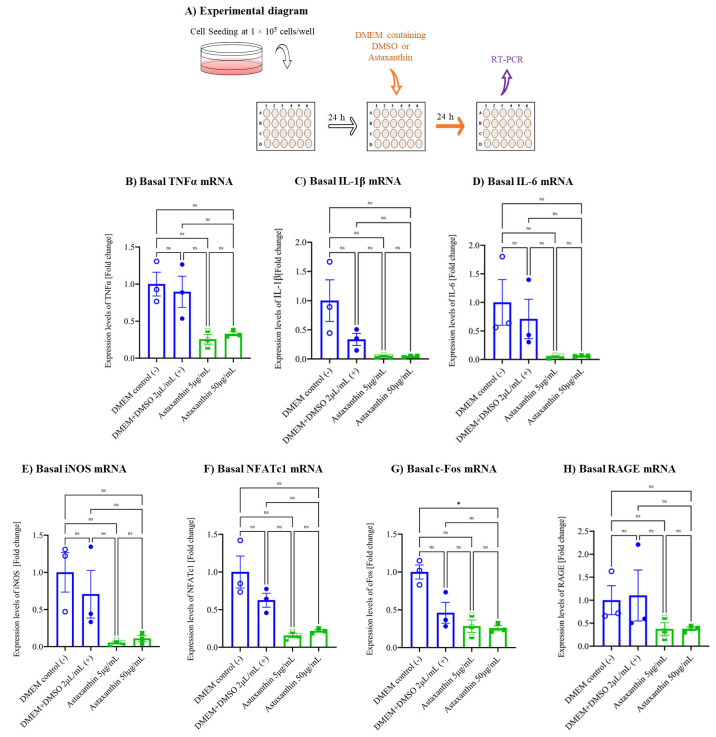
Effect of astaxanthin on autoinflammatory gene expression in RAW 264.7 cells. (**A**) Schematic representation of experimental procedures. Cells were seeded the same as for the inflammatory gene expression experiments and treated with astaxanthin or DMSO for 24 h, but with no further treatment with CML-HSA, to measure the autoinflammatory gene expression by qPCR. Expression was analyzed based on mRNA levels of (**B**) TNFα, (**C**) IL-1β, (**D**) IL-6, (**E**) iNOS, (**F**) NFATc1, (**G**) c-Fos, and (**H**) RAGE. All data were normalized using GAPDH and are shown as the mean ± SEM (*n* = 3) of the ratios against no treatment. * *p* < 0.05, ns denoted not significant by Tukey–Kramer test. DMSO, dimethyl sulfoxide; TNF, tumor necrosis factor; IL, interleukin; iNOS, inducible nitric oxide synthase; RAGE, receptor for advanced glycation end products; NFATc1, nuclear factor of activated T-cell, cytoplasmic 1; GAPDH, glyceraldehyde-3-phosphate dehydrogenase; SEM, standard error mean.

The cells were seeded in 24-well plates at a density of 1 × 10^5^ cells/well and incubated for 24 h. Afterward, cells were treated with astaxanthin or DMSO for an additional 24 h, which was followed by 0.5 μg/mL CML-HSA for 3 h (Figure 2A), or as stated in Figures 3A, 6A and 7A. Total RNA was extracted using Isogen II reagent (Nippon Gene, Toyama, Japan) according to the manufacturer’s instructions. RNase-free DNase-treated total RNA (500 ng) was used with PrimeScript RT Master Mix (Takara Bio Inc., Shiga, Japan). Applied Biosystems 2720 thermal cycler (Waltham, MA, USA) was used for reverse transcription. qPCR was performed using a Thunderbird SYBR qPCR mix (Toyobo Co., Ltd., Osaka, Japan) according to the manufacturer’s protocol and with gene-specific primers, as listed in Table 1 [59,61,63,64]. Briefly, the amplification reactions were conducted on an AB Applied Biosystems StepOnePlus real-time PCR system (Waltham, MA, USA). An initial hold step (95 °C for 1 min) and 40 cycles of PCR (95 °C for 15 s, 60 °C for 60 s), followed by generation of the dissociation curve. The comparative C_T_ method (2^(−ΔΔC^_T_^)^) was used to determine the fold change in target gene expression. Glyceraldehyde-3-phosphate dehydrogenase (GAPDH) was used for normalization.

### 2.9. F-Actin Ring Formation

**Figure 4:**
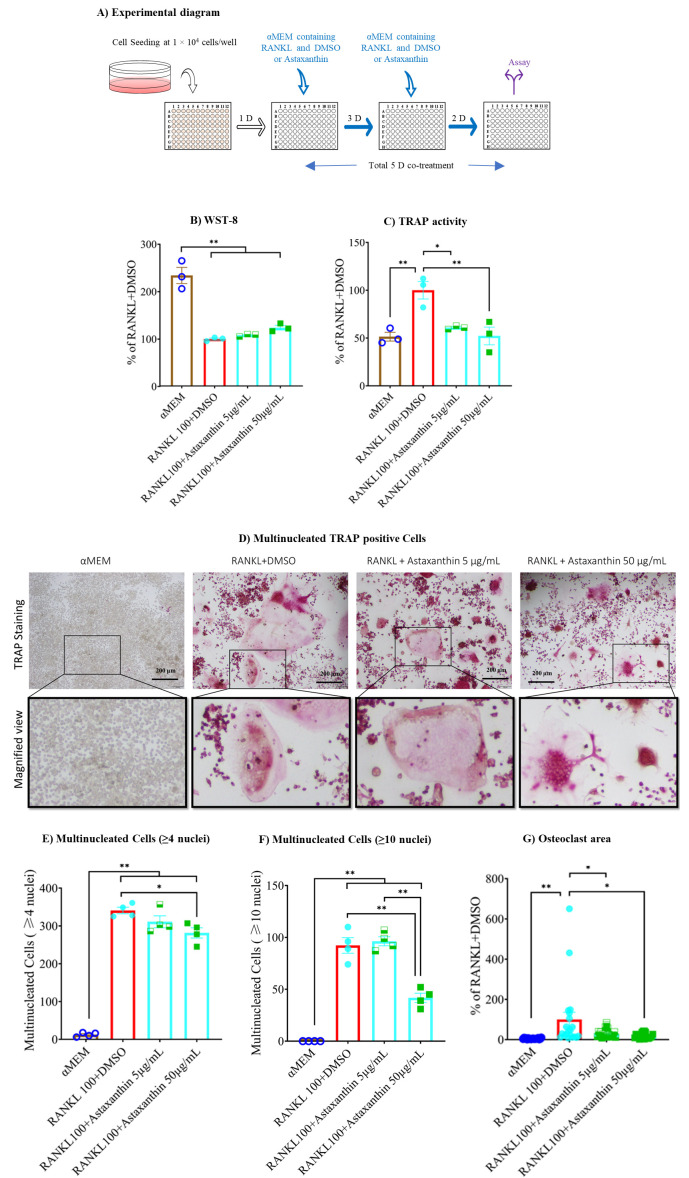
Effect of astaxanthin on cell viability, TRAP activity, and TRAP staining. (**A**) Schematic representation of experimental procedures. RAW 264.7 cells were plated in 96-well plates at 1 × 10^4^ cells/well. The next day, cells were treated with αMEM containing 10% FBS, and 100 ng/mL RANKL with or without DMSO or astaxanthin. After three days, the media were renewed. After five days, cells were used for (**B**) WST-8 assay, (**C**) osteoclastogenic TRAP activity assay, (**D**) TRAP staining for multinucleated TRAP positive cells, (**E**) multinucleated cell (≥4 nuclei) analysis, (**F**) multinucleated cell (≥10 nuclei) analysis, and (**G**) osteoclast area analysis. All data are shown as means ± SEM, * *p* < 0.05, ** *p* < 0.01, Tukey–Kramer test. The bar in the figure represents 200 µm.

RAW 264.7 cells were seeded at 1 × 10^4^ cells/well in 96-well black wall/clear bottom plates (Greiner Bio-One, Frickenhausen, Germany). After the osteoclastogenic experiment (Figure 4A), cells were fixed with 4% formaldehyde for 15 min at room temperature. Then, fixed cells were stained in the dark for 2 h with Phalloidin-iFluor 488 (ab176753, Abcam, Cambridge, UK), according to the manufacturer’s protocol. Cells were washed with phosphate-buffered saline (PBS) 3 times and incubated with 4′,6-diamidino-2-phenylindole (DAPI) (PureBlu #135-1303, Bio-Rad Laboratories, Berkeley, CA, USA) (1:1000) in Milli-Q water for 20 min in darkness. Finally, the cells were washed with Milli-Q water 3 times, and representative images were captured with a fluorescent microscope (IX71, Olympus, Tokyo). Images were processed and analyzed using ImageJ software.

### 2.10. Immunofluorescence Assay

RAW 264.7 cells were seeded 1 × 10^4^ cells/well in 96-well black wall/clear bottom plates. Media was changed after 24 h, and cells were stimulated with 100 ng/mL RANKL and treated with either DMSO or astaxanthin. After 20 min of treatment (Figure 8A), cells were fixed with 4% formaldehyde for 15 min at room temperature, followed by 0.2% Triton X-100 in PBS for 10 min to increase permeability. Afterwards, cells were blocked with 3% bovine serum albumin (BSA) in PBST for 1 h and incubated with primary antibody in blocking buffer against phospho-p65 (#3033, Cell Signaling Technology, Danvers, MA, USA) (dilution 1:1000) at 4 °C overnight. The next day, after 3 washes with PBST, donkey anti-rabbit secondary antibody conjugated with PE (sc-3745, Santa Cruz Biotechnology, Inc., Dallas, TX, USA) in blocking buffer was used and incubated at room temperature in the dark for 2 h. After 2 h, cells were washed with PBST 3 times and incubated with DAPI (1:1000) in MilliQ water for 20 min in the dark. Finally, the cells were washed with MilliQ water 3 times and representative images were captured with a fluorescence microscope (Olympus IX71). Images were processed and analyzed using ImageJ software.

### 2.11. Statistical Analysis

Graphs were prepared using GraphPad Prism 8. Data were expressed as mean ± standard error of mean (SEM). All statistical analysis were performed using Tukey–Kramer test for intergroup comparison in all the experiments using Excel add-ins. Differences were considered significant at a significance level of 5%.

## 3. Results

### 3.1. Neither Astaxanthin nor CML-HSA Produced Any Cytotoxicity

The RAW 264.7 cells were seeded at a concentration of 2.5 × 10^4^ cells/well in DMEM and after 24 h, the medium was renewed, with or without 5 or 50 µg/mL astaxanthin or 2 µL/mL DMSO, and incubated for another 24 h, and this was followed by the addition of CML-HSA at 0.5 µg/mL. Three hours later, WST-8 and LDH were assayed (Figure 1A). The data show that none of our samples induced cytotoxicity, in contrast to the control DMEM group. Instead, they exerted a stimulatory trend by inducing cell viability (Figure 1B,C). Henceforth, we performed our next experiments with these concentrations.

### 3.2. Astaxanthin Inhibits CML-HSA-Induced Inflammation and Autoinflammation in Cell Culture Model

Initially, the TNFα protein secreted by cells into the media was measured by ELISA and we found that TNFα protein secretion significantly induced in the vehicle control (CML-HSA) compared to the untreated group. Astaxanthin did not alter secretion, as compared to the vehicle control (Figure 2B). Hereafter, qPCR was used to examine the role of astaxanthin in the induction of inflammatory gene expression. The data reveal that CML-HSA induced the expression of the inflammatory genes *TNFα*, *IL-1β*, *IL-6*, and *iNOS* by 4-, 30-, 3-, and 4-fold, respectively, in the vehicle control compared to the untreated group; both concentrations of astaxanthin inhibited CML-HSA-induced inflammatory gene expression two-fold or more compared to the vehicle control (Figure 2C–F). TNFα protein secretion and mRNA expression were induced in the vehicle control, which has the capacity to stimulate osteoclast differentiation and proliferation in addition to activating the NFAT pathway; therefore, later we checked *NFATc1* and *c-Fos* gene expression (Figure 2G,H). The data reveal that *NFATc1* and *c-Fos* gene expression were stimulated in the vehicle control and were inhibited two-fold or more by both concentrations of astaxanthin. As an AGE, CML-HSA has a higher chance to stimulate the receptor for AGEs (*RAGE*) gene expression, but based on our data, we did not find any stimulation of *RAGE* expression in the vehicle control. However, treatment with astaxanthin inhibited the expression by two-fold compared to the vehicle control (Figure 2I) and could therefore be responsible for the reduction of inflammatory gene expression. These data indicate that astaxanthin may inhibit CML-HSA-induced inflammation in an in vitro cell culture model.

### 3.3. Astaxanthin Inhibits Autoinflammation in the Cell Culture Model

To evaluate the anti-inflammatory effect of astaxanthin on autoinflammation (Figure 3A), we analyzed the basal mRNA expression of TNFα, IL-1β, IL-6, iNOS, NFATc1, c-Fos, and RAGE without CML-HSA stimulation (Figure 3B–H). We found that vehicle control did not stimulate the expression of any of the analyzed genes compared to the untreated group. However, both concentrations of astaxanthin inhibited the gene expression by 3-, 4-, and 9–10-fold in the case of *TNFα*, *c-Fos*; *NFATc1*, *RAGE*; and *IL-1β*, *IL-6*, and *iNOS*, respectively. The inhibition was not statistically significant but because of a visible inhibitory trend, the data suggest that astaxanthin may inhibit autoinflammation in an in vitro cell culture model.

### 3.4. Astaxanthin Inhibits TRAP Activity without Altering Cell Viability

After determining the anti-inflammatory effect of astaxanthin, we checked its effect on osteoclast differentiation. The cells were seeded and next day treated with RANKL and DMSO or astaxanthin. After three days, the media were renewed, and after five days, WST-8 assay for cell viability and TRAP activity assay for osteoclast differentiation were conducted (Figure 4A). The WST-8 assay revealed no alteration in cell viability in response to astaxanthin or RANKL or DMSO (Figure 4B). The TRAP activity data revealed that in the vehicle control RANKL and DMSO significantly stimulated TRAP activity compared to the untreated group, and astaxanthin significantly inhibited TRAP activity in a dose-dependent manner, in contrast to the vehicle control (Figure 4C). The TRAP staining was done to check the effect of astaxanthin on the size of multinucleated osteoclasts, and we found that the number of cells with ≥4 or ≥10 nuclei were not significantly inhibited when using astaxanthin at a lower concentration (i.e., 5 μg/mL); however, at a higher concentration (i.e., 50 μg/mL), the sizes of both (i.e., osteoclast cells having ≥4 or ≥10 nuclei) were significantly inhibited by astaxanthin. Osteoclast size was also significantly reduced by astaxanthin in a dose-dependent manner (Figure 4D,G). These data indicate that by inhibiting the formation of small and bigger osteoclast cells with ≥4 and ≥10 nuclei, respectively, astaxanthin is inhibiting TRAP activity.

### 3.5. F-Actin Ring Size Was Reduced by Astaxanthin

**Figure 5:**
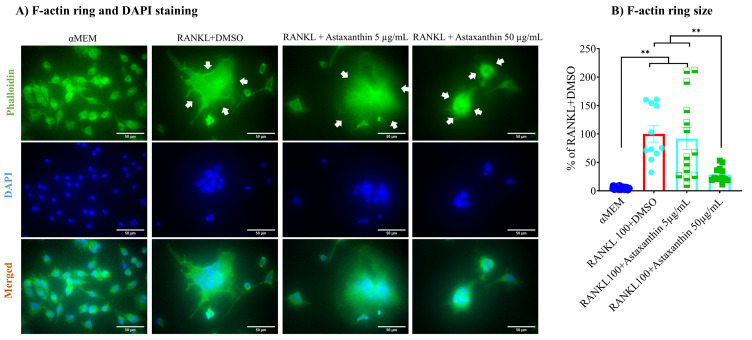
Effect of astaxanthin on F-actin ring size. (**A**) Cells were treated similarly to the TRAP activity experiment, which was followed by staining with phalloidin to stain cytoplasm as well as DAPI to stain nuclei. F-actin rings are indicated by white arrows in the figures. (**B**) F-actin ring size per osteoclast cells. All data are shown as means ± SEM, ** *p* < 0.01, Tukey–Kramer test. The bar in the figure represents 50 µm.

Multinucleated osteoclast cells are formed by the fusion of macrophages, and they form a transient resorption complex with F-actin rings and a ruffled border made of the plasma membrane that attracts other macrophages and even osteoclast cells, which then fuse to form giant multinucleated osteoclast cells. Resorption compartments create an acidic environment to degrade the bone matrix by proton pump and releasing cathepsin K, TRAP, and acid cysteine proteinase. Therefore, F-actin ring formation, size of the ring, and the number of nuclei per osteoclast play a vital role in osteoclastogenesis and bone resorption [62]. In our study, the formed F-actin ring was larger in size upon RANKL treatment of the vehicle control. The size was significantly inhibited by 50 µg/mL astaxanthin but was not reduced when lower concentration (i.e., 5 µg/mL) was used (Figure 5A,B). Together, these data suggest that astaxanthin reduces cell–cell fusion at sufficiently high concentrations.

### 3.6. Astaxanthin Inhibits Osteoclastogenic Gene Expression and NF-κB Pathway Activation

**Figure 6:**
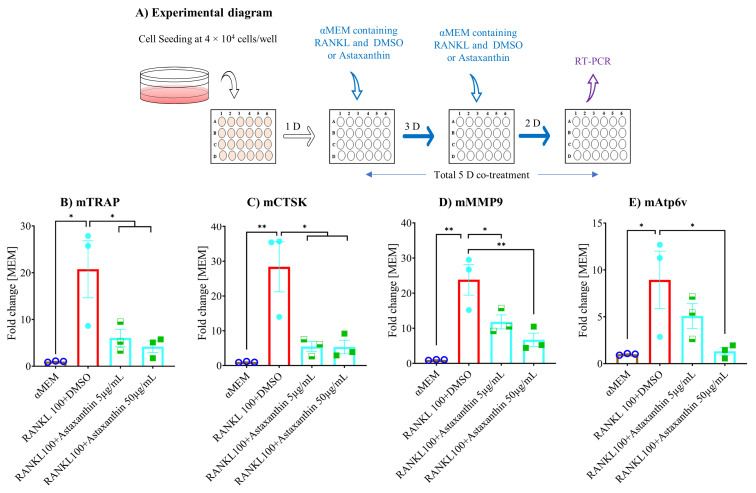
Effect of astaxanthin on osteoclastogenic maturation marker gene expression in RAW 264.7 cells. (**A**) Schematic representation of experimental procedures. RAW 264.7 cells were plated in 24-well plates at 4 × 10^4^ cells/well, and the next day, cells were treated with αMEM containing 10% FBS, 100 ng/mL RANKL with or without DMSO or astaxanthin. After three days, medium was renewed. After five days, the treated cells were collected, and mRNA was extracted. These were then used for cDNA synthesis and checked by qPCR. Expression was analyzed based on mRNA levels of (**B**) TRAP, (**C**) CTSK, (**D**) MMP 9, and (**E**) Atp6v were checked. Relative mRNA expression and data were normalized using GAPDH and are shown as fold-change. All data are shown as means ± SEM, *n* = 3. * *p* < 0.05, ** *p* < 0.01 by Tukey–Kramer test.

**Figure 7:**
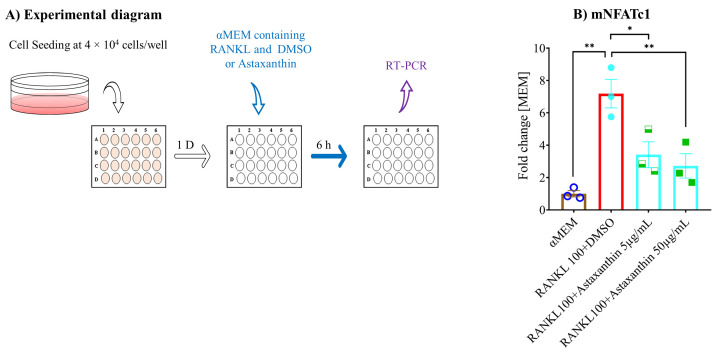
Effect of astaxanthin on osteoclastogenic early marker gene expression in RAW 264.7 cells. (**A**) Schematic representation of experimental procedures. RAW 264.7 cells were plated in 24-well plates at 4 × 10^4^ cells/well, and the next day, cells were treated with αMEM containing 10% FBS, 100 ng/mL RANKL with or without DMSO or astaxanthin. After six hours, the treated cells were collected, and mRNA was extracted. These were then used for cDNA synthesis and checked by qPCR. (**B**) mRNA expression of NFATc1. Relative mRNA expression, data were normalized using GAPDH and are shown as fold-change. All data are shown as means ± SEM, *n* = 3. * *p* < 0.05, ** *p* < 0.01, Tukey–Kramer test.

Furthermore, we used qPCR to determine the role of astaxanthin on osteoclastogenic gene expression, through which osteoclastogenesis is inhibited. The levels of osteoclastogenesis maturation marker genes *TRAP*, *CTSK*, *MMP9*, and *Atp6v* were analyzed after five days of treatment with or without astaxanthin (Figure 6A). The data showed that the expression of all analyzed genes was induced in the vehicle control in contrast to the untreated group (αMEM), and they were inhibited by astaxanthin in a dose-dependent manner compared to the vehicle control (RANKL+DMSO) (Figure 6B–E). Subsequently, we analyzed the expression of an early marker gene, *NFATc1*, which is considered the master regulator of osteoclastogenesis, and we found that after six hours of treatment with or without astaxanthin (Figure 7A), NFATc1 expression was induced significantly in the vehicle control compared to the untreated group and significantly inhibited by astaxanthin in a dose-dependent manner compared to the vehicle control (Figure 7B).

**Figure 8:**
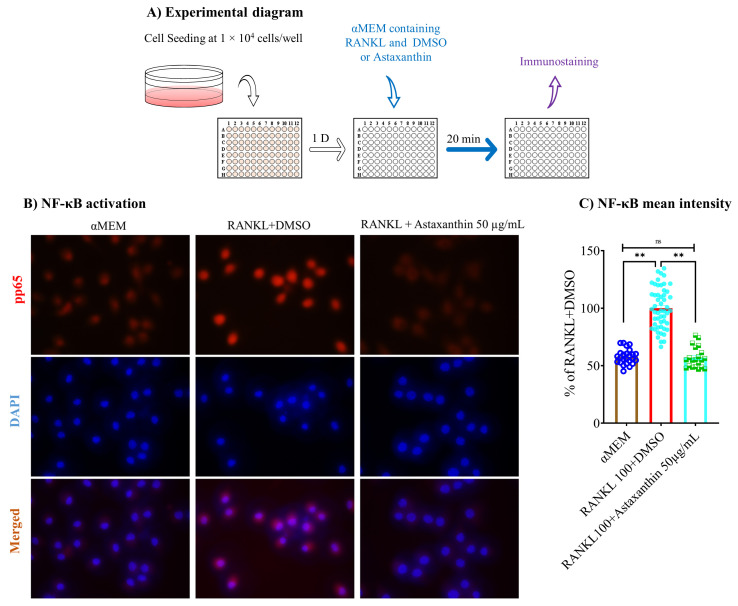
Effect of astaxanthin on NF-κB pathway activation. (**A**) Schematic representation of experimental procedures. RAW 264.7 cells were seeded in 96-well plates at 1 × 10^4^ cells/well. The following day, cells were treated with αMEM containing 10% FBS, 100 ng/mL RANKL with or without DMSO or astaxanthin. After 20 min of treatment, cells were stained for immunofluorescence assay. (**B**) Representative images of activated NF-κB and DAPI staining. (**C**) Activated NF-κB mean intensity. All data are shown as means ± SEM, ** *p* < 0.01, ns denoted not significant by Tukey–Kramer test.

NF-κB is one of the major targeted pathways that is activated after RANKL–RANK interaction. We studied the activation of NF-κB to check whether astaxanthin had inhibited its activation using immunostaining assay (Figure 8A). We found that 20 min of treatment with 50 µg/mL astaxanthin significantly inhibited activation of the NF-κB pathway (Figure 8B,C). The data reveal that the inhibition of osteoclastogenesis by astaxanthin occurs via the inhibition of NF-κB activation, which inhibits the expression of genes, required for osteoclastogenesis.

## 4. Discussion

Inflammation and osteoclast differentiation have always been linked due to certain cytokines being common in both phenomena. Increased osteoclast differentiation leads to diseases such as osteoporosis. Cytokines such TNFα, IL-1, and IL-6 are inflammatory, and if the inflammatory tissues are close to the bone surface, they induce bone loss by inducing osteoclast differentiation [6,17,65,66,67]. Astaxanthin is a carotenoid red pigment that is rich in biological properties. It has antioxidative, anti-inflammatory, and anticarcinogenic properties. The AGEs resulting from glycation reaction cause oxidative stress by producing ROS. Considering this, we explored the potential of astaxanthin as a candidate to reduce inflammation and glycative stress caused by oxidation. Previously, there have been reports that research-grade astaxanthin inhibited inflammatory and osteoclastogenic gene expression in vitro and in vivo [58,67,68,69]. However, to the best of our knowledge, no research on osteoclastogenesis and inflammation has been published regarding ASR, which is a commercially available astaxanthin supplement, except one of our pilot study where we have reported the inhibitory effect of ASR on TRAP activity [60]. In the present study, we analyzed the mechanism behind the inhibitory effect on osteoclastogenesis along with its effect against inflammation. Since there are numerous steps of isolation, purification, packaging, and storage while producing commercial-grade astaxanthin, we planned to check if there were any alterations in function due to those steps and henceforth, carried out the study to check the effect of astaxanthin in our in vitro model. We studied the effect of astaxanthin on CML-HSA-induced inflammatory gene expression and RANKL-induced osteoclastogenesis. We used the indicated concentrations of astaxanthin from the capsule formulation for our experiments. The exact concentration of CML-HSA in human blood is not currently known, but our previous study reveals that 0.5 µg/mL CML-HSA induced TNFα production in RAW 264.7 cells [40,41,61]. Our current data showed that astaxanthin inhibited both autoinflammatory and CML-HSA-induced inflammatory gene expression without altering cell viability. These data suggest that ASR or other formulations of active astaxanthin have the potential to be used as a supplement to inhibit inflammation resulting from CML-HSA and may reduce autoinflammation induced by cell–cell interactions.

**Figure 9:**
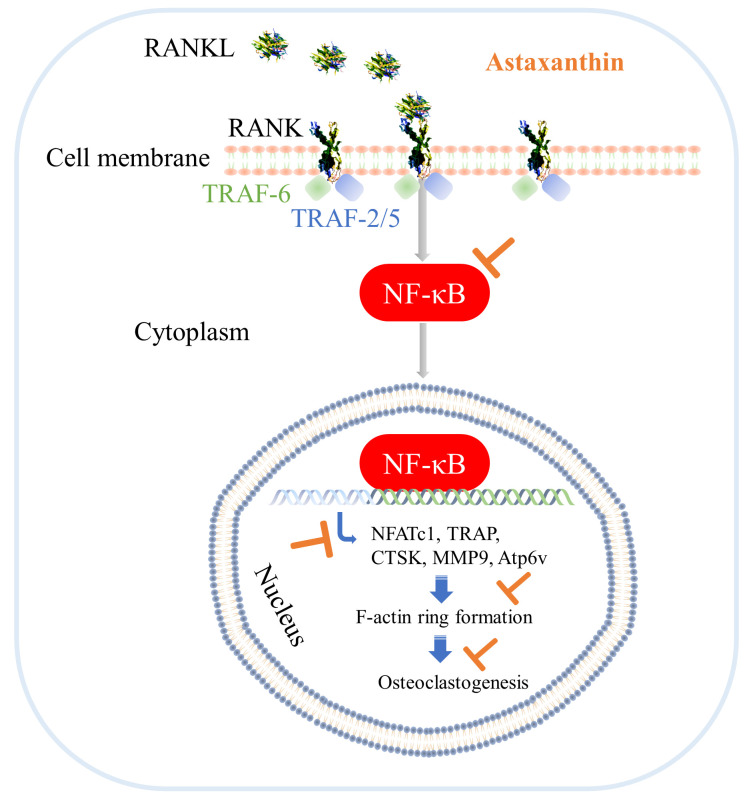
Schematic representation of the study summary. RANKL-induced osteoclastogenesis is significantly inhibited by astaxanthin via reductions in the activation of nuclear factor-κB (NF-κB), expression of NFATc1, multinucleated giant cell formation, and mature osteoclast marker gene expression.

A study was conducted on postmenopausal healthy women treated for 8 weeks with oral astaxanthin at a dose of 12 mg daily. The report revealed that astaxanthin may have the capacity to enhance antioxidation, reduce blood pressure, and decrease the vascular resistance in lower limbs [70]. Another study reported on the suppressive effect of astaxanthin on enteric flora cluttering in high-fat-fed (35% fat) mice [71], providing evidence for the beneficial effects of astaxanthin on human and animal health. Inflammatory bone diseases, such as RA, osteoarthritis, periodontitis, multiple myeloma, and metastatic tumors, to name a few, result in bone loss in the elderly [58]. Studies demonstrated that astaxanthin could reduce oxidative stress, inflammatory and osteoclastogenic cytokine production, and thereby play an important role in the restoration of bone homeostasis [72]. Astaxanthin treatment to Wister rats demonstrated a reduction in osteoclast cell count with an induction in osteoblast cell count along with a reduction in inflammation, suggesting that astaxanthin can improve bone health by reducing osteoclastic bone loss and osteoblastic bone formation [73]. Another study by Hoshi et al. has reported that astaxanthin has the capacity to restore the inhibited osteoblast differentiation in MC3T3-E1 cells treated with acetaldehyde and in bone marrow mesenchymal stem cells of transgenic mice expressing Aldh2*2 [74]. Excessive osteoclastic bone resorption is one of the primary causes of bone loss. Osteoclasts originate from monocyte/macrophage lineage and possess RANK and RAGE receptors on their surfaces. The RANK ligands bind with RANK receptors and regulate downstream signaling for NF-κB-pathway activation. Several ex vivo studies have revealed that TNFα promoted RANKL-induced osteoclastogenesis through NF-κB activation by mediating p13K/AKT signaling [6,7,17,18,19,65,75]. Our present study demonstrated that astaxanthin could inhibit osteoclastogenesis by reducing osteoclastogenic gene expression and cell-cell fusion (Figure 9) that is similar to the previous study by Yun et al. using research grade astaxanthin, [58] providing evidence that the astaxanthin in the capsule is bioactive, and functional.

## 5. Conclusions

ASR possesses bioactive astaxanthin, and preparations using ASR showed a significant inhibitory effect on in vitro osteoclastogenesis when used in treatments at higher concentrations in addition to an inhibitory trend on CML-HSA-induced and autoinflammatory gene expression.

## Figures and Tables

**Table 1:** Primer sequences.

Primer Name	Forward	Reverse
TNFα	ACC CTC ACA CTC AGA TCA TCT TC	TGG TGG TTT GCT ACG ACG T
IL-1β	TGT AAT GAA AGA CGG CAC ACC	TCT TCT TTG GGT ATT GCT TGG
IL-6	ACA ACC ACG GCC TTC CCT ACT T	CAC GAT TTC CCA GAG AAC ATG TG
iNOS	CCA AGC CCT CAC CTA CTT CC	CTC TGA GGG CTG ACA CAA GG
NFATc1	GGA GCG GAG AAA CTT TGC G	GTG ACA CTA GGG GAC ACA TAA CT
TRAP	GCG ACC ATT GTT AGC CAC ATA CG	CGT TGA TGT CGC ACA GAG GGA T
RAGE	ACT ACC GAG TCC GAG TCT ACC	GTA GCT TCC CTC AGA CAC ACA
c-Fos	CGG GTT TCA ACG CCG ACT A	TTG GCA CTA GAG ACG GAC AGA
CTSK	GAA GAA GAC TCA CCA GAA GCA G	TCC AGG TTA TGG GCA GAG ATT
Atp6v0	ACG GTG ATG TCA CAG CAG ACG T	CCT CTG GAT AGA GCC TGC CGC A
GAPDH	AGG TCG GTG TGA ACG GAT TTG	TGT AGA CCA TGT AGT TGA GGT CA
MMP9	CTG GAC AGC CAG ACA CTA AAG	CTC GCG GCA AGT CTT CAG AG

## Data Availability

Not applicable.
